# Variation in Morphological Characters of Two Invasive Leafminers, *Liriomyza huidobrensis* and *L. sativae,* across a Tropical Elevation Gradient

**DOI:** 10.1673/031.011.6901

**Published:** 2011-05-28

**Authors:** Warsito Tantowijoyo, Ary A. Hoffmann

**Affiliations:** Centre for Environmental Stress and Adaptation Research, Department of Zoology, University of Melbourne, Parkville, Victoria 3010, Australia

**Keywords:** colonization, elevation, genetic differences, phenotypic plasticity

## Abstract

Changes in morphological traits along elevation and latitudinal gradients in ectotherms are often interpreted in terms of the temperature-size rule, which states that the body size of organisms increases under low temperatures, and is therefore expected to increase with elevation and latitude. However other factors like host plant might contribute to spatial patterns in size as well, particularly for polyphagous insects. Here elevation patterns for trait size and shape in two leafminer species are examined, *Liriomyza huidobrensis* (Blanchard) (Diptera: Agromyzidae) and *L. sativae* Blanchard, along a tropical elevation gradient in Java, Indonesia. Adult leafminers were trapped from different locations in the mountainous area of Dieng in the province of Central Java. To separate environmental versus genetic effects, *L. huidobrensis* originating from 1378 m and 2129 m ASL were reared in the laboratory for five generations. Size variation along the elevation gradient was only found in *L. huidobrensis* and this followed expectations based on the temperature-size rule. There were also complex changes in wing shape along the gradient. Morphological differences were influenced by genetic and environmental effects. Findings are discussed within the context of adaptation to different elevations in the two species.

## Introduction

In insects, the size of morphological traits often changes along elevation and latitudinal gradients (Blanckenhorn and Demont 2004; Hodkinson 2005). The size of traits is often positively correlated with elevation and latitude (e.g., Alonzo 1999; Malo and Baonza 2002; Smith et al. 2000), consistent with patterns expected under the temperature-size rule, which states that the body size of ectotherms increases with low temperatures (Atkinson 1994). However the opposite pattern can also occur (e.g. Brehm and Fiedler 2004; Kubota et al. 2007; Sota 1996) and trait size variation might not be correlated with elevation or latitude (Hawkins and DeVries 1996; Hawkins and Lawton 1995).

Along elevation gradients, insects face different environmental conditions that could alter body size either directly through plastic effects or indirectly as a result of selection pressures changing with environmental conditions (Mousseau and Dingle, 1991; Baldwin 1999; Blanckenhorn and Demont 2004;). Size variation can influence fitness traits like development and reproduction (Berger et al. 2008; Gotthard et al. 2007; Harvey 2005; Larsson and Kustvall 1990; Tammaru et al. 2002), sexual and somatic development (Blanckenhorn 2006; Etile and Despland 2008; Fischer et al. 2003), and thermoregulation (Bishop and Armbruster 1999; Kingsolver et al. 2004; Kingsolver and Koehl 1985; Kingsolver and Watt 1983). Size variation can also influence dispersal ability (Gutierrez and Menendez 1997; Hoffmann et al. 2007). These factors could drive local size differentiation along environmental gradients. However changes in trait size might also simply reflect temperature effects along elevation and longitudinal gradients on the rate of growth and development time of insects (Hodkinson 2005). These environ mental effects are often not separated from genetic changes in size along a gradient by rearing insects from different parts of the gradient in the same environment.

Insects can show elevation and latitudinal patterns in trait shape as well as size. In *Drosophila serrata,* there is a latitudinal gradient for wing shape that involves genetically based changes in the length of the wing relative to its width (Hoffmann and Shirriffs 2002). These patterns for wing shape appear to be at least partly independent of changes in wing size. However in another *Drosophila* species, *D. mediopunctata,* wing does not vary along a thermal gradient, in contrast to genetically-based changes in wing size (Bitner-Mathé et al. 1995; Bitner-Mathé and Klaczko 1999). At present, there are no clear hypotheses about changes in shape along gradients to guide predictions, although a common observation in *Drosophila* is that wing length increases relative to wing width at low temperatures (Azevedo et al. 1998; Loh et al. 2008).

In this study, we test whether elevation patterns are present for trait size and trait shape in tropical populations of the invasive pest leafminers, *Liriomyza huidobrensis* (Blanchard) (Diptera: Agromyzidae) and *L. sativae* Blanchard from Java, Indonesia. The rate of growth and development time of these species vary inversely with temperature (Lanzoni et al. 2002; Tokumaru and Abe 2003) and the species are also likely to differ in resistance to temperature extremes (Parrella 1987). *L. huidobrensis* is more cold resistant than *L. sativae*, although it is still capable of developing under warm conditions (Huang et al. 2007; Tokumaru and Abe 2003). The distribution of *L. sativae* and *L. huidobrensis* varies with elevation in Central Java (Tantowijoyo and Hoffmann 2010). *Liriomyza sativae* predominantly colonizes hosts below 600 m ASL (above sea level), while *L. huidobrensis* predominates above 1000 m and is the only species collected at 1400 m ASL.

To date morphological studies on leafminer species have tended to focus on morphological variation as a method for identifying species (Shiao 2004), or to assess the effect of host crops on development (Videla et al. 2006) rather than focusing on spatial patterns. However, leafminers provide good systems to study morphological variability in the tropics because they can be collected in large numbers and reared in common environments to separate environmental and genetic effects, and also because they colonize a wide range of elevations.

Three questions were addressed. (1) Do *L. huidobrensis* and *L. sativae* that colonize different elevations vary in size and does this variation follow predictions from the temperature-size rule? (2) Are there any changes in wing shape including wing aspect along elevation gradients? (3) Do elevation differences in morphology have a genetic and/or environmental basis? Variation in morphological characters of the leafminer species was assessed by measuring thorax length, wing size, wing shape, and wing aspect. To determine whether differences in these traits between elevation extremes were genetic, measurements were also made on samples reared in a common laboratory environment for five generations.

## Materials and Methods

### Field sampling

Adults were trapped with a test tube (diameter by length: 1.8 cm by 15 cm) in the morning (0630–1100 hours) or afternoon (1500–1800 hours) when they were inactive. Adults were trapped between 74 m and 2166 m ASL in the mountainous area of Dieng, in the province of Central Java, Indonesia, between latitudes 6.9519° S and 7.4111° S and longitudes 109.4528° E and 109.9931° E. Sampling sites were separated by elevations of around 100 m, with 33 sites in total. Up to 10 females and males from each elevation were randomly selected for morphological measurements.

Individuals were trapped from different hosts at the different elevations. At 200 m ASL they were mostly obtained from cucumber (*Cucumis sativus*), long bean (*Vigna sinensis*), and choy sum (*Brassica rapa*); at 400–1000 m they were collected from cucumber, long bean, and snap bean (*Phaseolus vulgaris*); and above 1000 m they were collected from potato (*Solanum tuberosum*), faba bean (*Vicia faba*), snap bean, and red bean (*V. sinensis*). During the survey, temperature was recorded with Hobo (www.onsetcomp.com) data loggers every 2 hours that were placed at around 200 m elevation intervals. The data loggers were placed in plastic vials (diameter by height, 7 cm by 7 cm) with 6–8 holes of 1.0 cm diameter for air ventilation and two additional holes in the bottom of the vials to prevent moisture accumulating. The vials were attached to bamboo sticks and placed 1 m above ground level in farmed fields within crops, just above crop foliage that extended up to 1 m (except for beans grown on trellises).

### Laboratory rearing

Rearing took place from October 2006 to January 2007 at Bogor Agriculture University, Indonesia. Temperature was not controlled, but ranged throughout the culture period from 20 to 31° C due to daily fluctuations (there was no consistent increase or decrease in temperature). A photoperiod of 14:10 L:D was used.

Colonies for rearing were initially obtained from potato foliage that was infested at Jatilawang village (elevation = 1378 m ASL, longitude/latitude = 109.4555, -7.1250) and Sembungan village (elevation = 2129 m ASL, longitude/latitude = 109.5516, -7.1408). These sites represent the lowest and highest sites where *L. huidobrensis* could be consistently collected (*L. sativae* was not considered in these experiments because there was little elevation-related size variation — see below).

In the laboratory, both colonies were separately maintained for five generations on choy sum. The colonies were fed with a 10% honey solution provided on cotton hung inside the cage and replaced every three days. In each generation, offspring were obtained from the first and second oviposition periods of 3 days; these were measured and used to establish populations for the next generation. Ten offspring were measured from each generation of culture.

### Morphological measurement

Thorax and wing traits were measured and used to represent variation in total body size. Based on Honek's assumption (Honek 1993), the size of various parts was assumed to vary isometrically and variation in size of distal or proximal body parts was assumed to be equal to the variation of the total body length. For thorax measurement, flies were placed in a ventral view, and thorax length was measured from the anterior margin of the thorax to the tip of scutellum using a graticule. Wings were measured on the same flies following a published procedure (Shiao 2004) and after mounting them on a microscope slide with double sided sticky tape.

**Figure 1.**
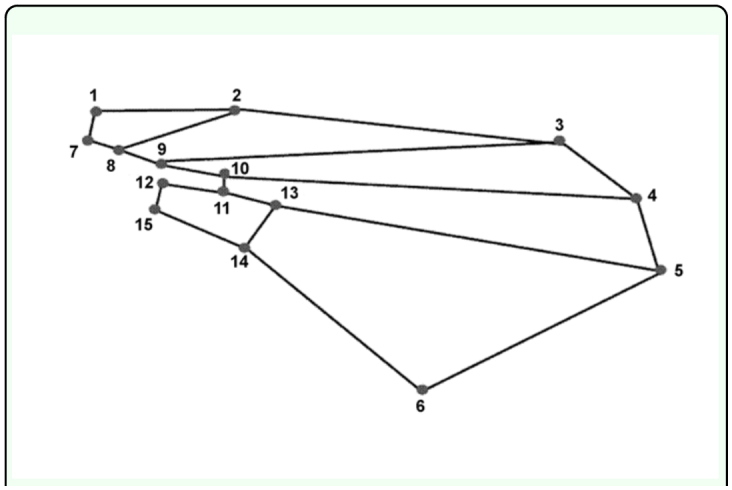
Positions of 15 landmarks on the wing of females and males of *L. huidobrensis* and *L. sativae* used for wing measurements. High quality figures are available online

Images of wings were captured with a Pixelink (www.pixelink.com) digital camera. The wing from only one side of each adult was measured. A series of landmarks were obtained from each wing (Figure 1) using TPSDig version 1.31 (http://life.bio.sunysb.edu/morph). Landmarks were digitized after wings were rotated to face the same direction. Since the venation forming landmark number 15 was unclear, only 15 landmarks were used in this study compared to 16 measured by Shiao (2004). Landmarks were measured three times and averaged to decrease measurement variation. Coordinates were then used to compute centroid size, and individual coordinates were also converted to Procrustes coordinates using TPSRe1W version 1.24 (http://life.bio.sunysb.edu/morph) for shape analysis (below). Wing centroid is calculated as the square root of the sum of the squared distances of each landmark from the centroid (Bookstein, 1991; Kolliker-Ott et al., 2003) that provides a measure of overall wing size.

### Analysis

Statistical analyses were performed in SPSS version 15. Thorax length and wing size, data were first grouped into 200 m elevation categories because we were interested in changes in trait variances as well as means along the gradient. Differences in thorax length and wing size between elevation categories were then assessed by elevation, using one-way ANOVAs. In addition, linear regressions were undertaken to test whether size measures changed continuously with elevation.

To investigate whether morphological variation (as opposed to changes in means) varied with elevation, homogeneity tests on coefficients of variation using the Miller and Feltz approach (Zar 1996) were carried out. For *L. huidobrensis,* this comparison involved data from the lowest, middle, and highest elevations (due to sample size variation, different elevations were used for males and females, see below). For *L. sativae*, all three or four samples collected were compared (this species was collected from a more limited elevation range - see below).

The Procrustes procedure (Klingenberg and McIntyre 1998) was used to test whether varation in wing shape depended to elevation. Procrustes coordinates were first computed for each wing to correct for differences in size, position, and orientation. Using the TPSRe1W 1.24 program (Rohlf 2007), this analysis followed two steps: superimposing all wings in a particular comparison (e.g. elevation for a particular species/sex) to form the same size and then rotating their configurations around the centroid to produce the optimal fit of corresponding landmarks. The effects of elevation and individual on wing shape variation were then assessed. Procrustes coordinates were analyzed through nested ANOVAs using SPSS. Instead of using degrees of freedom (*df*) from conventional nested ANOVAs, *df* were computed by multiplying the number of *df* from a univariate ANOVA by two times the number of landmarks minus four (see Klingenberg and McIntyre 1998). The Procrustes sum of squares were summed across landmarks and divided by the computed *df.* The last stage was to determine the relative importance of different landmarks on shape effects of elevation as outlined in Klingenberg and McIntyre (1998), by computing variance components associated with the x- and y-Procrustes coordinates of each landmark. The x- and y-sums of square were added together and variance components were then assessed.

These variation components were presented as percentages.

To describe movements of each landmark in field samples, mean shape of the wing of different sexes and species originating from highest and lowest elevations were compared to mean shape of all samples collected across elevations (as a control), and then visualized through the thin-plate spline transformation grid using *R* version 2.6.0 (http://www.r-project.org/ 2007). In this visualization, a square grid on mean shape of wing originating from a particular elevation was deformed smoothly using a pair of thin-plate splines to a curved grid on the mean shapes of the wing of controls. However, a comparison of mean shape of wing female and male *L. huidobrensis* was taken using individuals originating from 1200 m and 2200 m ASL The low elevation site represented the lowest point at which this species could be consistently collected. Deformation of wing shape was oriented in three directions: (a) along the x-axis which corresponds to antero-posterior axis, (b) along the y-axes which corresponds to the dorso-ventral axis, and (c) equally along the x- and y-axis when the deformation of landmarks have equal magnitude (Zelditch et al. 2004). Every direction has positive and negative orientations.

A common change in wing shape considered in the literature is wing aspect, the extent to which the width of the wing changes relative to its length (Azevedo et al. 1998; Hoffmann and Shirriffs 2002; Tanaka et al. 2007). To test whether this wing shape pattern varied with elevation, wing aspect was analyzed using two-way ANOVAs with elevation and sex as fixed factors. Sex was involved in the analysis to assess any sex based dimorphism. Wing aspect was scored as wing width (the linear distance between landmarks 3 and 6) divided by wing length (the linear distance between landmarks 5 and 7). Thus, a smaller wing aspect indicates a more elongated wing.

The above analyses were also undertaken with leafminers reared under laboratory conditions. In this case, two-way ANOVAs were undertaken to test for differences in thorax length, wing size, and wing aspect with generations and field origins as fixed factors. The analysis included the field sample of flies that was considered as generation 0. Regressions of thorax length and wing size of each field origin against generation were also undertaken to test for changes in size over time due to laboratory adaptation. Wing shape changes as well as size changes were examined. The thin-plate spline transformation grid was performed on the first and the fifth generations of females cultured in the laboratory.

## Results

### Field samples

For the morphological analysis, around 200 *L. huidobrensis* and 70 *L. sativae* of each sex were measured. Results of two-way ANOVAs indicate that for thorax length there were significant (all *p* < 0.001, with *df* = 1, 488) effects of sex (F = 1.338), species (F = 491.626), and their interaction (F=18.240). For wing size, significant effects (all *p* < 0.001, with *df* = 1, 497) were also detected for sex (F = 368.436), species (F = 2781.790), and their interaction (F = 132.869). For both traits, *L. huidobrensis* was always significantly bigger than *L. sativae* and females were always bigger than males. For *L. huidobrensis* females and males, thorax length averaged 0.78 (± 0.0035) mm and 0.72 (± 0.0036) mm, whereas those of *L. sativae* averaged 0.63 (± 0.0073) mm and 0.61 (± 0.0073) mm, respectively. Wing size of *L. huidobrensis* averaged 2.53 (± 0.0098) mm and 2.10 (± 0.010) mm, whereas those of *L. sativae* were 1.63 (± 0.0172) mm and 1.52 (± 0.0204) mm for females and males, respectively.

**Table 1.**
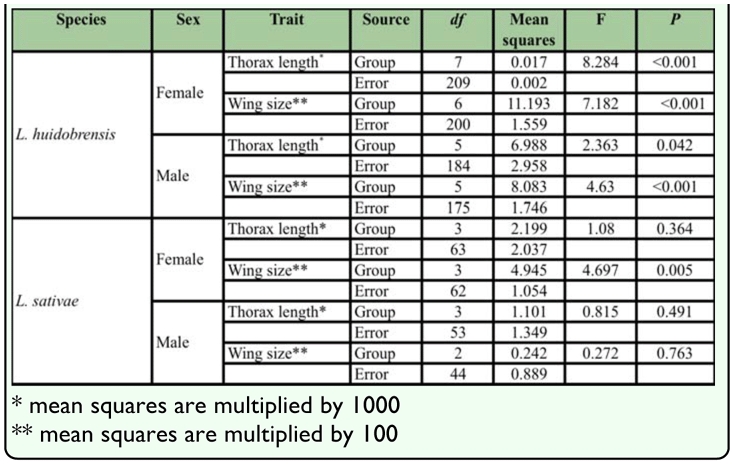
Results of one-way ANOVAs testing differences between elevation sites for thorax length and wing size (in mm) of female and male *Liriomyza huidobrensis* and *L. sativae.*

**Figure 2.**
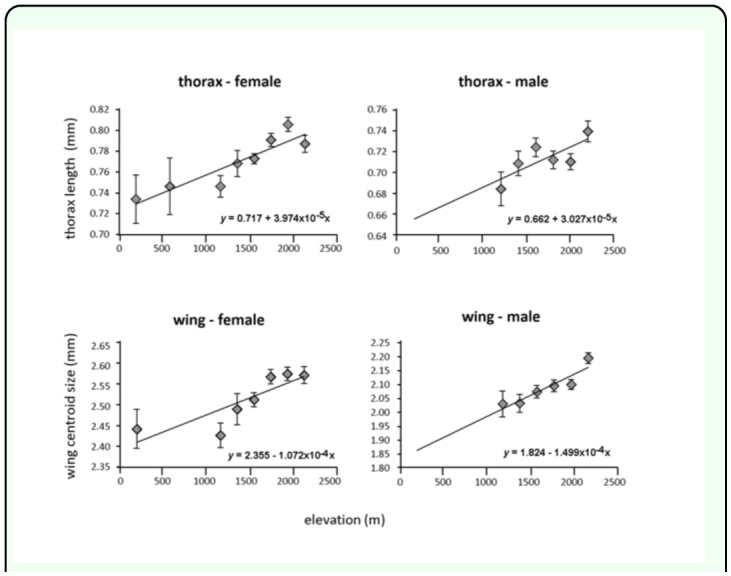
Thorax length and wing centroid size of female and male *Liriomyza huidobrensis* plotted against elevation. Values are means ± standard errors. Linear regression lines and equations predicting size (y) from elevation (x) are shown on the graphs. High quality figures are available online

Results of one-way ANOVAs show that thorax length and wing size of both male and female *L. huidobrensis* varied significantly among sites (Table 1). The regression analyses indicate that the variation of thorax length and wing size significantly increased with elevation (Figure 2), with significant regressions for thorax for females (F = 35.70, *df* = 1, 222, *p* <0.001, R^2^ = 0.14) and males (F = 4.54, *df* = 1, 188, *p* = 0.034, R^2^ = 0.02), and regressions for wing also were significant for females (F = 30.27, *df* = 1, 205, *p* < 0.001, R^2^ = 0.13) and males (F = 18.273, *df* = 1, 179, *p* < 0.001; R^2^ = 0.09).

Levels of variation for thorax length and wing size among individuals tended to be similar at the three selected elevations compared (200, 1400, 2200 m ASL for females and 400, 1400, 2200 m ASL for males) except for female thorax length. The homogeneity indexes of Miller and Feltz varied significantly for female thorax length (*χ*^2^ = 7.663, df = 2, *p* = 0.022), but not for female wing size (*X*2 = 2.518, *df* = 2, *p* = 0.284), male thorax length (*χ*^2^ = 0.525, *df* = 2, *P* = 0.769), or male wing size (*χ*^2^ = 5.558, *df* = 2, *p* = 0.062). Regressions showed that the female thorax length variation was significantly associated with elevation (F = 16.00, *df* = 1, 6, *P* = 0.007, R^2^ = 0.71) and variation decreased with elevation (v = 9.948-0.03*x*). Even though site differences were not significant for female wing size, the regression suggested that variation in this trait also decreased with elevation (F = 6.58, *df =* 1, 5, *p* = 0.051, R^2^ = 0.57).

**Table 2.**
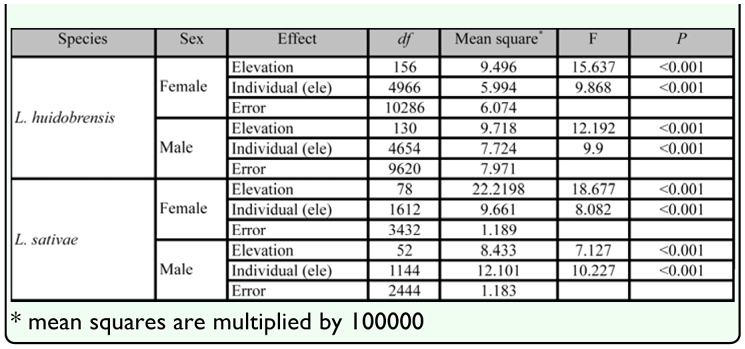
Procrustes ANOVAs on wing shape measurements of field female and male *Liriomyza huidobrensis* and *L. sativae.* The effects of elevation and individual (nested within elevation) were assessed.

**Figure 3.**
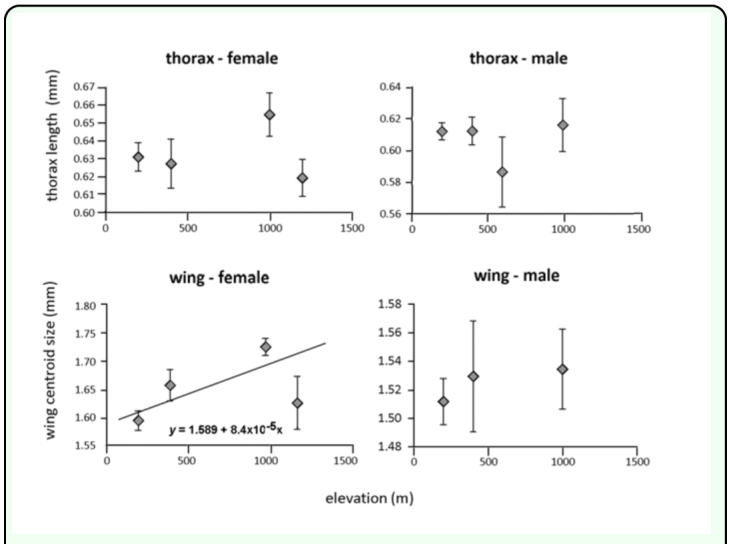
Thorax length and wing centroid size of female and male *Liriomyza sativae* at different elevations. Values are means ± standard errors values. Linear regression lines and equation predicting size (y) from elevation (x) are shown on one graph where a significant association was detected. High quality figures are available online.

In contrast to *L. huidobrensis,* thorax length and wing size of *L. sativae* did not vary among sites, except in the case of female wing size (Table 1). Female wing size was associated with elevation in the regression analysis (F = 6.138; *df* =1, 64; *p* = 0.0159, R^2^ = 0.09) and increased at high elevations (Figure 3) but there were no obvious patterns for thorax length. Data from 200 m, 400 m and 1000 m ASL were used to compute Miller and Feltz homogeneity indexes and these varied among sites for male thorax variation (*χ*^2^= 8.032, *df* = 3, *P* = 0.045). However, the coefficient of variation did not change monotonically with elevation, but was relatively higher in the middle of the gradient compared 200 m and 1000 m ASL.

**Figure 4.**
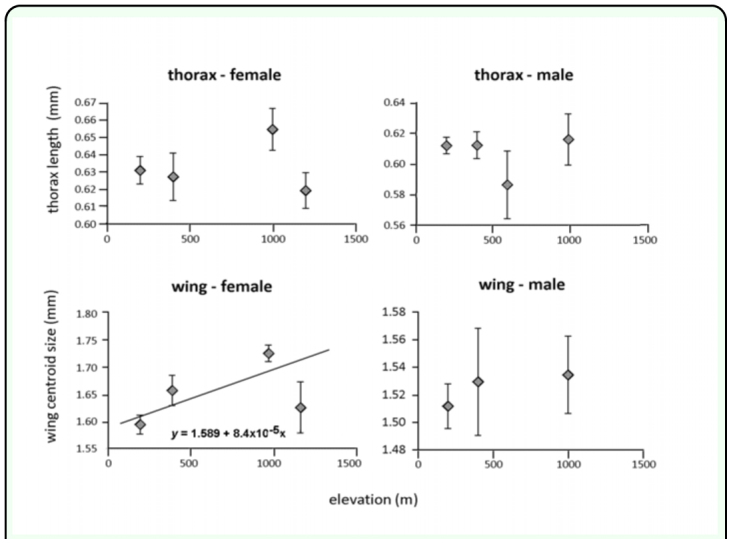
Variance components (expressed as a percentage) of (a) elevation and (b) individual nested within elevation for the different landmarks of *Liriomyza huidobrensis* and *L. sativae* females. Variance components were obtained from Procrustes analysis as described in the text. Each bar represents a single landmark. Bars with a relatively high percentage indicate that the landmark varied substantially among elevation sites or individuals. High quality figures are available online

Wing shape of females and males of both species varied among elevations and among individuals (Table 2). For females, variance components associated with elevation were generally higher for *L. sativae* than *L. huidobrensis,* with the variation in shape mostly due to landmarks 3, 10, 11, and 13, where elevation represented 11.7%, 13.7%, 11.5%, and 10.2% of the variance (Figure 4). For female *L. huidobrensis,* variance components due to elevation were mostly less than 5% for the different landmarks. For the among individual effect (nested within elevation), variation of wing shape in both species was shared among landmarks and highest percentages were found for landmarks 3, 4, 5, 6, 10, 11, 13, and 14 where the individual effect accounted for around 70% of the variance (Figure 4).

For males, different landmarks contributed to the effect of elevation on variation in wing shape in *L. huidobrensis* and *L. sativae.* Variance components for individuals were similar across landmarks in the two species. For *L. huidobrensis,* landmarks 6, 10, and 11 were predominantly involved in shape changes with elevation. For these landmarks, elevation represented 5.5%, 5.5%, and 4.9% of the variance. In contrast for *L. sativae,* landmarks 12 and 14 were particularly important and represented around 10% of the variance. As in the case of females, shape variation among individuals for both species was shared across the landmarks.

**Figure 5.**
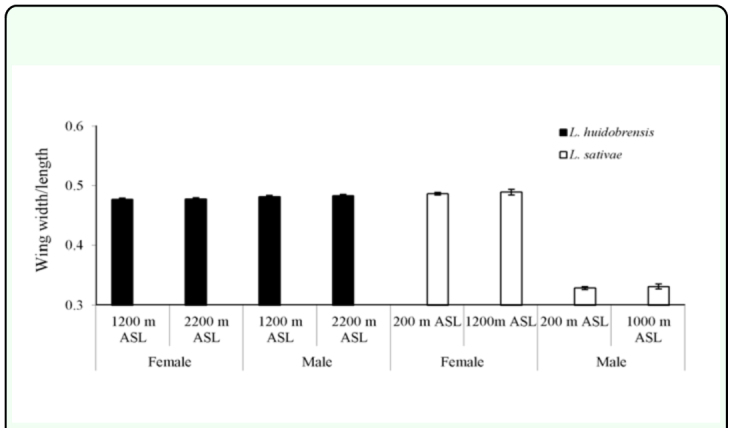
Wing aspects of female and male *Liriomyza huidobrensis* and *L. sativae* originating from the lowest and highest elevations. Values are means ± standard errors. High quality figures are available online

Mean shape of females and males were compared through thin-plate spline transformation grids on individuals originating from different elevations. However, for neither species did we see consistent changes in shape with elevation, despite the fact that shape varied among the elevation samples. Moreover, deformations of wing landmarks in individuals originating from the lowest and highest elevation did not change wing aspect. In both species, wing aspect was not influenced by elevation, but was influenced by sex (*L. huidobrensis*: F = 5.645, *df* = 1, 98, *p* = 0.019 and *L. sativae*: F = 1935.449, *df* = 1.79, *p* < 0.001). The means for high and low elevation sites of the two species are plotted in Figure 5. At both locations wing aspect of female *L. huidobrensis* is reduced compared to males, whereas female *L. sativae* show a strikingly larger wing aspect than males.

### Laboratory comparison

**Figure 6.**
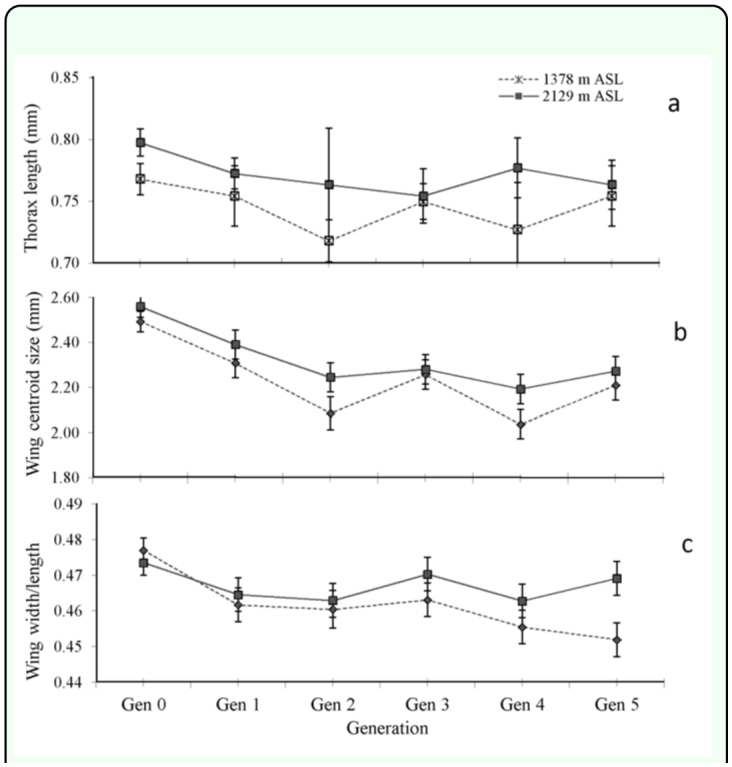
Changes of (a) thorax length, (b) wing centroid, and (c) wing aspect in female *Liriomyza huidobrensis* originating from two different elevations over generations. Values are means ± standard error values. High quality figures are available online

The laboratory temperatures where rearing was carried out were warmer than those encountered at the high elevation field sites; the average temperature was 23.4° C (range 20.0 to 31.0° C) compared to 19.5° C (range 6.0° C to 39.5° C) at 1522 m ASL and 13.6° C (range 4.5° C to 37.0° C) at 2166 m ASL. Two-way ANOVAs were used to test for environmental (generation) and genetic (field origin) contributions to elevation differences in morphology of female *L. huidobrensis* between the 1378 m and 2129 m ASL sites. The analyses indicate that thorax length did not differ across generations, even though field samples as well as the five generations of laboratory rearing were included in the data (Table 3). In contrast, the mean thorax length of the flies differed among elevations; flies from the low elevation site were consistently smaller than those from the high elevation site (Figure 6). A Miller and Feltz homogeneity test indicated that only variability in thorax length of individuals originating from 2129 m ASL varied among generations (*χ*^2^ = 3.193, *df* = 5, *p* = 0.022), but there was no consistent pattern across generations by regression (F = 0.107, *df* = 1, *4, p* = 0.760) and variation was highest in the second generation of laboratory rearing.

**Table 3.**
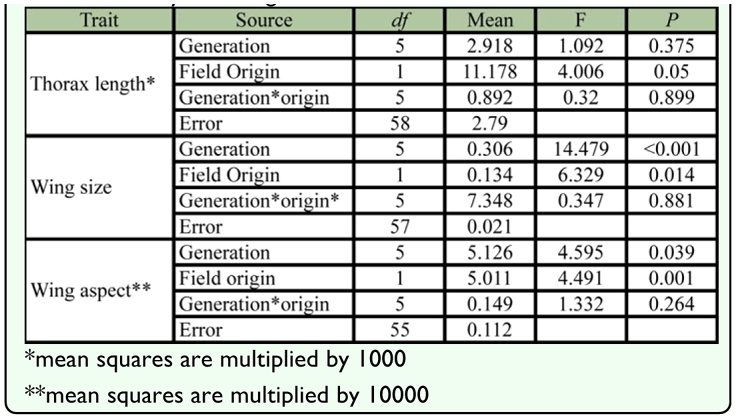
Results of two-way ANOVAs on thorax length, wing size and wing aspect of female *Liriomyza huidobrensis* reared in the field or in the laboratory for 1–5 generations.

In contrast to thorax length, wing size changed with environmental conditions in the laboratory (Table 3). In the first generation, wing size was significantly reduced compared to field samples, and this difference was maintained across subsequent generations (Figure 6). Regression of wing size in both colonies (excluding the field sample) showed no change with generation (1378 m: F = 1.046; *df* = 1, 22, *p* = 0.318 and 2129 m: F = 1.876; *df* = 1, 23, *p* = 0.184). A significant difference in size between individuals originating from 1378 m and 2129 m ASL was only found in the second and the fourth laboratory generations, but the high elevation colony was always larger than the low elevation colony. The Miller and Feltz homogeneity test results indicate that only wing size of individuals originating from 2129 m ASL varied with generation (*χ*^2^ = 12.062, *df* = 5, *p* = 0.034), but regression analysis showed no consistent pattern across generations (F = 0.08, *df* = 1, 4 *p* = 0.791). As for thorax length, the second laboratory generation showed the highest level of variation.

Wing shape of the two colonies varied among generations and individuals (Table 4). For females from 1378 m colony, variance components due to generation were mostly associated with landmarks 4 and 5. The variance components of these landmarks due to generation were 31.8% and 30.8%, respectively. On the other hand, the highest variance components due to generation on females from the 2129 m colony were shared by landmarks 13 and 14, accounting for 27.9% and 24.4%, respectively. The thin-plate spline analysis showed that deformation of wing landmarks of reared individuals was complex compared to the field sample and also tended to differ between colonies (results not presented). Wings of female *L. huidobrensis* originating from 1378 m ASL became relatively elongated, whereas those of *L. huidobrensis* originating from 2129 m became wider at the base.

**Table 4.**
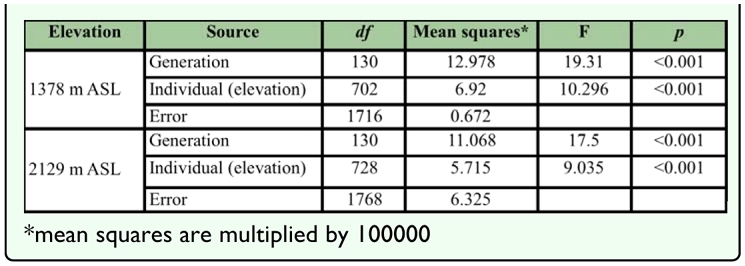
Procrustes ANOVAs on wing shape measurements of female *Liriomyza huidobrensis* originating from 1378 m and 2129 m ASL on generations.

Consistent with the deformation of landmarks, wing aspect of females originating from 1378 m and 2129 m ASL differed with elevation and changed across generations (Table 3). Wing aspect in the 2129 m ASL colony was relatively larger, indicating that females from this location had relatively wider wings than those from 1378 m. However this pattern was not evident in the field samples (Figure 6). Wing aspect only tended to decrease after rearing in the laboratory, particularly for the low elevation colony (Figure 6).

## Discussion

Patterns of morphological variation along the elevation gradient differed between the species. With a wider range of elevations colonized, thorax length and wing size of female and male *L. huidobrensis* varied with elevation and followed predictions from the temperature-size rule, whereas there were no consistent patterns for *L. sativae.* These species differences may simply reflect the fact that *L. huidobrensis* colonized a wider range of elevations (and associated climatic conditions) than *L. sativae.* The *L. sativae* colonization area was limited to the range 0–1200 m ASL where host composition was relatively similar consisting only of cucumber and bean crops. The differences in median and minimum temperatures in this elevation range were 6 and 8° C respectively. On the other hand, *L. huidobrensis* was found at all elevations (0–2200 m ASL) where host crop composition varied from cucumber and beans at low elevations to potato and faba beans at high elevations. Differences in median and minimum temperatures between the lowest with highest elevations reached 10 and 12° C respectively, and more data points were available for establishing trends.

Thorax length and wing size were quite variable in *L. huidobrensis.* In females, the difference between the smallest and the largest individual at any elevation for thorax length was 0.3 mm (37.7% of the overall mean thorax length). For size it was 1.0 mm (37.8%). In males, the equivalent figures were 0.3 mm (41.3%) for thorax length and 0.8 mm (40.0%) for size. This varation in size may influence physiological traits. For instance Willmer and Unwin (1981) found that body size was strongly correlated with heat gaining capacity in larger insects. Large leafminers may be at an advantage at low temperatures, but could also have a decreased fitness when radiation levels are high. Conversely, with a relatively rapid heat loss, smaller leafminers may be favored under warm conditions. However this needs to be tested directly by carrying out stress tests on flies with different sizes.

In *L. huidobrensis*, variation in traits along elevation gradients may be explained by the effects of temperature and hosts on development rather than through direct selection on size. Even though no study has assessed the direct effect of low temperature on body size of *L. huidobrensis,* an increase of thorax length and wing size is expected at lower temperatures. The slower development time of *L. huidobrensis* in colder temperatures might increase energy gain that then increases body size (Davidowitz and Nijhout 2004; Etile and Despland 2008; Kingsolver et al. 2004; Lanzoni et al. 2002). Differences in host composition along elevation gradients might also cause an increase of thorax length. As shown by Videla et al. (2006), *L. huidobrensis* developing on the most favorable hosts (faba bean and potato) can be larger than those that emerge from beans and other crops including cucumber, *C. maxima.* This might contribute to an elevation-related difference in size because faba bean and potato crops predominate at high elevations.

However genetic factors also appear to contribute to the size differences. The thorax size of *L. huidobrensis* did not change after rearing for five generations under laboratory conditions, and flies derived from a high elevation site were consistently larger than those from a low elevation site. Moreover, the parallel decline in wing size in both the high and low elevation colonies of this species suggests that wing size responds to selection, in this case through selection imposed by the laboratory environment. Nevertheless, the laboratory rearing experiment also points to environmental effects, as indicated by the initial decrease in wing size when flies were transferred from the field to the laboratory. This effect might partly reflect rearing host given that leafminers originated from potato, red beans, and/or faba beans in the field, whereas in the laboratory they were maintained on choy sum. However the laboratory conditions where rearing was undertaken were warmer than those experienced at high elevations, and it seems likely that at least part of this response is related to temperature.

The greater variation of thorax length among female *L. huidobrensis* collected from low elevations might reflect host effects, variability in density, effects of selection, or other factors like microclimate variability and interactions between temperature and hosts. Low elevation sites are likely to represent a novel habitat for *L. huidobrensis* (Rauf et al. 2000). High elevation sites with colder temperatures are favored by *L. huidobrensis* (Chen et al. 2003; Huang and Kang 2007; Rauf et al. 2000; Spencer and Steyskal 1973; Zhao and Kang 2000). At these sites, hosts were less heterogeneous and consisted mainly of potatoes.

Variance components for landmarks and thin-plate spline analyses revealed variation in wing shape between samples in both species. Variation was greater in *L. sativae* compared to *L. huidobrensis* and the types of landmarks involved differed between the species. The pattern of landmark deformation across the elevation gradient was complex and did not follow simple patterns as for wing size. Moreover, changes in wing size of *L. huidobrensis* along the elevation gradient were not correlated with changes in shape or wing aspect. In *L. sativae,* there were changes in wing shape even though wing size did not vary with elevation. These results suggest that shape variation responds in a different way to environmental factors and perhaps also genetic factors. Previous research has suggested that in insects wing shape is particularly responsive to environmental variation (Hoffmann et al. 2005).

Overall, *L. huidobrensis* were larger than *L. sativae* even when collected at the same elevation. The difference in body size might influence the colonization ability of these species following Hutchinson and MacArthur (1959) who suggested that smaller species tend to be specialized with a narrower host and environmental range when compared to larger species. In the case of the two leafminer species, the smaller *L. sativae* colonized a narrower elevation range than the larger *L. huidobrensis.* Larger body size in *L. huidobrensis* might provide higher heat resistance and cold tolerance compared to *L. sativae* (Huang and Kang 2007). Insects with larger body size have a better ability to conserve energy and this can increase their survival when they experience low temperature (Renault et al. 2003). Being larger also benefits an insect's ability to resist desiccation when dealing with warmer condition (Le Lagadec et al. 1998). Finally, larger body size may benefit movement rates, particularly if insect have relatively longer wings relative to their body (Gutierrez and Menendez 1997; Hoffmann et al. 2007).

In conclusion, wing and thorax size varied along an elevation gradient in *L. huidobrensis,* but not in *L. sativae.* The size variation in *L. huidobrensis* followed Bergmann's rule and appears to be influenced by genetic factors, although environmental effects are also important particularly for wing size. Variation in wing shape of *L. sativae* was greater than in *L. huidobrensis*, but wing shape changes did not fall into patterns that could be easily related to elevation. More work is needed to test patterns of selection on size and associations between movement rates and size in leafminers.
